# The Development of Transformative Agency of Teachers in Teaching Research Activities in China

**DOI:** 10.3389/fpsyg.2021.724175

**Published:** 2022-01-20

**Authors:** Chunting Diao, Xuan Zhou, Qiming Mao, Jianzhong Hong

**Affiliations:** ^1^School of Psychology, Key Laboratory of Adolescent Cyber Psychology and Behavior, Central China Normal University, Wuhan, China; ^2^School of Humanities, Hubei University of Chinese Medicine, Wuhan, China; ^3^School of Mechanical Engineering, Wuhan Polytechnic University, Wuhan, China; ^4^School of Education, Central China Normal University, Wuhan, China

**Keywords:** activity theory, change laboratory, elementary school teacher, teaching research activities, transformative agency

## Abstract

Teaching research activities (TRA) in China are practical and reflective research of teachers on teaching. These required activities are meant to ensure quality education and facilitate the professional development of teachers. However, in TRA, teachers encounter many challenges such as low efficiency and weak team collaboration. These problems make it hard to achieve the expected outcomes. *W Primary School* reformed its activities using Change Laboratory, a formative intervention approach to workplace learning and development based on activity theory. The data collected included seven recorded meetings in the Change Laboratory. The conversations in the meetings were then transcribed into texts. A deductive method of content analysis was used to code the data, focusing on categorizing comments of teachers about the transformative agency. The findings showed the following: (1) There were five types of transformative agencies, namely, resisting, criticizing, explicating, envisioning, and committing to actions. Resisting and criticizing were represented less frequently, and taking action did not emerge as a type of transformative agency. (2) The comments about transformative agency about tools were more frequent than comments about other elements in the activity system. (3) There were some differences in the expression of transformative agency across participants. At the end of this study, the implications for the development of TRA are discussed.

## Introduction

Teaching research activities (TRA) in China are practical and reflective research of teachers on teaching. The method originated from the former Soviet Union and gradually developed its Chinese characteristics. Now, it has become a regular activity for primary school teachers in China. TRA include implementation of curriculum plans, collective preparation for teaching, conducting school-based research activities, and creating open teaching classrooms (Gong, 2014). In recent decades, educational change agents have been trying to facilitate pedagogical innovations among teachers since the implementation of the New Curriculum Reforms in 2001. Under the New Curriculum Reforms of China, “teachers are researchers,” and the professional development of teachers is increasingly emphasized. Teachers are expected to critically reflect on their activity to improve and develop their teaching practices.

Teaching research activities are assumed to be crucial for ensuring the quality of education of children as well as for facilitating the professional development of teachers. However, teachers have pointed out that they encounter many problems such as low efficiency and weak team collaboration. These problems make it hard to achieve the intended outcomes. Based on the literature, there appear to be three aspects of these problems. First, TRA are from top to down, usually carried out within the framework prescribed by a superior educational administrative department, which makes them lack flexibility in content and form (e.g., Chen, 2012; Li, 2013). Second, the top-down selection of the topics for TRA prioritizes “advanced and novel” topics but ignores practical applications of ideas; TRA do not address the problems that teachers consider most urgent to be solved (e.g., Zhang and Zhao, 2007). Third, the subjectivity of teachers in evaluating TRA and disagreements among teachers about how to accomplish TRA has not been given much attention (e.g., Tan and Fan, 2017).

The transformative agency is essential to TRA. It exists in wider communities and work settings (Engeström, 2015). Based on the activity theoretical framework, the transformative agency has been defined as “breaking away from the given frame of action and taking the initiative to transform it; it is the capacity to form and implement intentions that go beyond and transform the accepted routines and given conditions of an activity” (Virkkunen, 2006; Vänninen et al., 2015). Transformative agency fosters joint activities among teachers as they explain and envision new possibilities through collective interaction over time (Haapasaari et al., 2016). It may be expressed in discourse and understood as a specific type and instrumentality of language-mediated organizational action, in which the participants express and transform a goal (Hall and Seidel Horn, 2012). It can promote change in organizations, and the purpose of TRA is to promote change in the teaching practices.

Change Laboratory (Engeström et al., 1996; Virkkunen and Newnham, 2013) is a formative intervention approach to workplace learning and educational change that builds on activity theory. The method was first implemented in 1995 in Finland. Since then, it has been implemented in work organizations ranging from hospitals and schools to factories and office settings of different countries (Engeström, 2015). It aims at developing generative solutions over long periods in both research activities and the research communities (Sannino et al., 2016). The results of a Change Laboratory are initially local and their diffusion typically takes place as further experimentation, development, and enrichment rather than as direct transfer and multiplication of the created solutions. Change Laboratory provides potential new ways of working that can be envisioned, designed, experienced, and experimented on by a small team through intensive discussion (Virkkunen and Newnham, 2013). A Change Laboratory intervention typically consists of 6 to 12 weekly sessions that last for about 2 h each. In the Change Laboratory sessions, participants, and researcher-interventionists use a set of representational devices designed for jointly analyzing disturbances and contradictions in their activities and for developing new solutions (Sannino et al., 2016). The emergence and evolution of transformative agency are considered an important outcome of the Change Laboratory (Haapasaari et al., 2016). Change Laboratory interventions can purposefully facilitate transformative agency (Virkkunen and Newnham, 2013). In our research, Change Laboratory is an intervention that helps teachers go through a process of change in their sense of transformative agency and change their skills to enact a new sense of transformative agency. The Change Laboratory supports the transformative agency of teachers and helps us to observe and understand transformative agency through discussions with the participant teachers.

### Transformative Agency of Teachers in Teaching Research Activities

Some researchers point out that the practical participation of teachers in TRA and even leading the process have significant meaning for the growth of teachers (Villegas-Reimers, 2003; Potari, 2013; Gong, 2014). Self-determinism also makes the subjectivity of teachers meaningful. Ryan and Deci (2000) emphasized that when the behavior of an individual is more autonomous, he or she will recognize the role of oneself in transformation more and treat it as important. Accordingly, researchers have also suggested that the motivation and the individual initiative of teachers to participate in activities should be internalized and enhanced (Mushayikwa and Lubben, 2009; Ho et al., 2021). Participation is a central characteristic in situated activity, and it is learning within the practices of the community. Teachers also experience personal development as they engage in these practices (Lave, 1991; Yamagata-Lynch and Haudenschild, 2009). The transformative agency of teachers usually emerges in collective discussions about the classroom community. When teachers create interactional spaces for breaking away from the traditional “taken-for-granted” patterns of activities, they can attain transformative agency (Lipponen and Kumpulainen, 2011; Engeström, 2015). Regarding subjectivity of teachers, since most current TRA are designed and carried out by school management in China, participant teachers are often passive agents of policies and reforms. This may make teachers lack the initiative to participate in TRA. Although many studies have pointed to the important role of participant teachers in change (Sannino, 2010; Gong, 2014), there are limited studies that analyze the change of individual motivation in these activities (Thurlings and den Brok, 2017). Moreover, we should pay attention not only to individual participants but also to their transformative agency, which includes the interconnection and complexity of the local TRA system.

### Research on Transformative Agency With Activity Theory

Activity theory (Engeström, 2008, 2015) explains human activity as an object-oriented, culturally mediated system with six interconnected components: subject, object, tools, community, rules, and division of labor. It aims to explain the collective, transformative processes as part of a complex activity system. In an activity system, tools reflect Vygotsky’s concept of mediation. They are needed as “auxiliary stimuli” to facilitate breaking away from the problematic or conflicting situation and overcoming “the pull of the past.” They can be anything used in the transformation process (Jonassen and Rohrer-Murphy, 1999; Sannino, 2014).

The existing literature on the transformative agency is mostly theoretical, mainly explaining or discussing transformative agency based on activity theory and/or Change Laboratory interventions (Virkkunen, 2006; Haapasaari and Kerosuo, 2015; Tuominen and Lehtonen, 2018). One researcher proposed the concept of radical-transformative agency as a struggle against inequality, economic oppression, racism, and other forms of injustice related to the historical and political contexts of the world-historical struggle (Stetsenko, 2019). A few empirical studies have analyzed expressions of transformative agency as part of the activity system (Vänninen et al., 2015; Haapasaari et al., 2016). Accordingly, some specific types of transformative agencies manifested in talk, such as resisting, criticizing, explicating, envisioning, committing to actions, and taking actions, have been identified (Sannino, 2010; Haapasaari et al., 2016). One study analyzed transformative agency as the quality of expansive learning; in this sense, the transformative agency can be pursued through formative interventions that break away from the confines of the researchers as instructional intentions of interventionists (Sannino et al., 2016). These ideas illuminate transformative agency in developing TRA as an interactive and collective effort that should be examined in a dynamic way (Lund and Eriksen, 2016).

### Chinese Culture and the Expression of Transformative Agency

The Change Laboratory and the discussions with teachers in this study were carried out in China, and this means the characteristics of Chinese communication must be considered. The dominant culture in China is collectivistic. When people express their opinions, they will first consider the feelings of others, and they often use euphemisms or keep silent (Morrison, 2011; Chen, 2012; Tan and Fan, 2017). At the same time, Chinese culture is a typical high-context communication culture, in which most of the information is either in the physical context or is internalized in the person (Hall, 1976). For this reason, too, the expression of transformative agency would be influenced by culture. This issue is something we return to when we discuss our analysis of the Change Laboratory as it was applied to understanding the transformative agency of teachers.

The case study and the Change Laboratory were carried out in W Primary School, located in a city in central China. We analyzed dialogs among teacher participants to gain insight into the transformative agency of teachers. The data collected include seven recorded meetings in the Change Laboratory. They were then transcribed into texts and coded with a categorization of transformative agency expressions (Haapasaari et al., 2016). The questions of interest were as follows: How is the transformative agency of primary teachers expressed and how does it develop in the Change Laboratory discussion of TRA? The more specific aspects covered in these dialogs were (1) the development of transformative agency of teachers in different stages, (2) the expression of transformative agency about the activity system, and (3) differences in the transformative agency across participants.

## Materials and Methods

We conducted a Change Laboratory intervention in *W Primary School*. The contents, materials, and stimulating tools of every session were planned by the researchers in their roles as interventionists. The participants discussed how to change the content and form of current TRA to make them more efficient. We examined whether teachers showed new expressions of transformative agency and increases in transformative agency throughout these discussions. This was a qualitative and quantitative study. Transcripts of the group discussions were analyzed qualitatively to examine the experiences of teachers with TRA and the emerging expressions of transformative agency. The transcripts were also analyzed quantitatively to assess change in the number and types of expressions of transformative agency over time.

### Participants

The Laboratory participants were young teachers of the school and university researchers. The school is located in a suburb of Wuhan, China. Most students of this school were children of migrant workers, who were not highly educated and had less time and resources to spend on the education of their children than other parents. This meant that teachers invested more time and energy in the education of students than would be the case in other schools. Another feature of the school was the large proportion of young teachers. Because young teachers are more energetic and open than older teachers, the idea of school management was to let the young teachers be the vanguard of reform. Upon the agreement of school management and based on the needs of teachers, our research group proposed the experiment of using Change Laboratory to reform the TRA. A total of ten teachers were selected to participate in the Change Laboratory, considering the spare time of teachers and subjects. Among them, three teachers were replaced after Session 1 because of low participation and an expressed unwillingness to participate in the following meetings.

As shown in Table 1, the final teacher participants included one man and nine women with a mean age of 26 years. Eight of them had bachelor’s degree and two had master’s degree. They taught a range of subjects: Chinese (five teachers), Math (two teachers), English (two teachers), and Physical education (one teacher). The teachers taught students from grades 2 to 5. The length of their teaching experience ranged from 0.5 to 6 years, with a mean duration of 1.8 years. In China, the Chinese teacher is usually the headteacher, managing all the affairs of one class. Therefore, the Chinese teacher usually leads only one class. The Math teacher leads two classes. The English teacher leads three classes, and the PE teacher leads eight classes. In W Primary School, the workload of teachers is 14 lessons per week for Chinese and Math teachers and 18 lessons for English and PE teachers. Each lesson lasts 40 min.

**TABLE 1:** The basic information of teacher participants.

Name	Gender	Age	Education background	Subject	Grade	Length of teaching	Workload (lessons per week)
Hui	Female	26	Bachelor	Chinese	4	2	14
Lin	Female	28	Master	Chinese	5	2	14
Ting	Female	27	Bachelor	Chinese	4	2	14
Chen	Female	28	Master	Chinese	5	1	14
Xin	Female	23	Bachelor	Math	2	2	14
Lee	Female	30	Bachelor	English	5	6	18
Ming	Female	24	Bachelor	English	5	1	18
Fu	Male	22	Bachelor	PE	4	0.5	18
Yi	Female	24	Bachelor	Chinese	3	2	14
Huan	Female	28	Master	Math	5	0.5	14

In the Change Laboratory, participants also included six researchers from a university. One of the researchers had a dual role in the project, as the long-time educational expert of the school and researcher of the Change Laboratory. Two researchers controlled the process of the Change Laboratory, including the preparation of stimulus tools and schedule. Another three researchers recorded and videotaped the sessions.

### Data Collection

The data collected in this study include seven discussion sessions held by teachers and researchers by Change Laboratory. These sessions were conducted during November 2018 and January 2019 and were videotaped with the consent of all the participants.

Before every session, a meeting agenda was prepared to ensure that the study procedure was followed, including how to promote the continuation of the discussion from the previous session. In the first two sessions, we noticed that the contributions of teachers were rather short, formal, and polite. The discussions were not very active, perhaps, because teachers did not know each other well and the researchers were new to them. To ease communication, starting with Session 3, we played an icebreaker game before each meeting, which seemed to help.

The discussion session lasted on an average of 126 min (shortest = 102 min; longest = 153 min); the total length of the seven sessions was 854 min. The videotapes were then transcribed verbatim. During transcription, the researchers strived to maintain the original meaning by completely and accurately recording the words spoken, the pauses, and the tone of the interviewees. The transcript contained a total of 1,914 speaking turns.

### Data Analysis

A deductive content analysis was adopted to analyze the data using the conceptual framework of transformative agency suggested by Haapasaari et al. (2016). In this framework, there were six categories of transformative agency, namely, *resisting*, *criticizing*, *explicating*, *envisioning*, *committing to action*, and *taking action*. Deductive content analysis (usually of text data but also paintings, songs, videos, etc.) divides information into meaningful categories and then describes the categories statistically. This method is also called quantitative analysis of qualitative data (Penlington, 2008; Krippendorff, 2018).

As a first step in the analysis, we used the transcripts to identify the six types of the transformative agency listed by Haapasaari et al. (2016) and counted the number of each type. There were 310 expressions of transformative agency in the transcribed data. They were coded according to the types of transformative agency expressions. We selected a speaking turn as a unit of analysis. Some speaking turns contained the necessary information for coding. Some of the speaking turns were difficult to interpret, and these were discussed by the researchers to reach an inter-coder agreement. The six expression types of transformative agency, with respective illustrative excerpts from the data, are presented in Table 2.

**TABLE 2:** Six types of the expressions of transformative agency.

Type of expression	Identification criteria
Resisting	Resisting the change, new suggestions or initiatives. Directed at management, co-workers or initiatives.
Criticizing	Criticizing the current activity and organization. Change-oriented and aiming at identifying problems in current ways of working.
Explicating	Explicating new possibilities or potentials in the activity. Relating to past positive experiences or former well-tried practices.
Envisioning	Envisioning new patterns or models in the activity. Future-oriented suggestions or presentations of a new way of working.
Committing to actions	Committing to taking concrete new actions to change the activity. Commissive speech acts tied to time and place.
Taking action	Taken consequential actions to change the activity between or after the laboratory sessions.

As a second step, we took the activity system as a dimension of analysis. The contents of the speaking turns were coded according to the six components of the activity system: subject, object, tools, community, rules, and division of labor. As some speaking turns contained two or more components of the activity, the total number was 332.

As a third step, we focused on the individual differences and change in transformative agency throughout the sessions. We recorded the frequency with which each participating teacher expressed each of the six types of transformative agency.

## Results

### The Development of Transformative Agency

In the analysis of participant expressions of transformative agency, we found mainly five of the six types as proposed by Haapasaari et al. (2016), namely, *resisting*, *criticizing*, *explicating*, *envisioning*, and *committing to actions*. *Taking action* did not occur because the Laboratory sessions lasted only 3 months, which was not enough time for the whole process of the reform of TRA in *W Primary School*.

The expression frequencies in each session are presented in Table 3. There were 310 expressions in total. The most frequent expression was *envisioning*, which numbered 119. The next was *explicating* new possibilities or potential in the activity, which numbered 104. The other categories were not frequent. There were 50 expressions of *criticizing*, 24 of *resisting*, and 13 of *committing to actions.*

**TABLE 3:** The expression types of transformative agency.

Session	Resisting *f(%)*	Criticizing *f(%)*	Explicating *f(%)*	Envisioning *f(%)*	Committing to actions *f(%)*	Taking actions *f(%)*	Total *f(%)*
1	4 (10.26)	18 (46.15)	13 (33.33)	4 (10.26)	0	0	39 (100)
2	6 (12.5)	12 (25)	25 (53.08)	5 (10.42)	0	0	48 (100)
3	8 (11.43)	7 (10)	28 (40)	27 (38.57)	0	0	70 (100)
4	1 (2.22)	7 (15.56)	11 (24.44)	21 (46.67)	5 (11.11)	0	45 (100)
5	1 (3.7)	1 (3.7)	6 (22.22)	13 (48.15)	6 (22.22)	0	27 (100)
6	0	0	4 (13.33)	25 (83.33)	1 (3.33)	0	30 (100)
7	4 (7.84)	5 (9.8)	17 (33.33)	24 (47.06)	1 (1.96)	0	51 (100)
Total	24 (7.74)	50 (16.13)	104 (33.55)	119 (38.39)	13 (4.19)	0	310 (100)

*Committing to actions* is committing to taking concrete, new actions to change the activity. In our Change Laboratory, teachers mainly explored reform options and then reported them to school leaders. Further action required the consent of school leaders, so there was less commitment to actions. Aside from *committing to actions*, the least frequent type of expression was *resisting*. The type of transformative agency usually takes the form of rejecting the suggestions from other participants or sharing their negative experiences. *Criticizing* was also expressed at low frequency, although it most often highlights the need for a change in the activity (Haapasaari et al., 2016). In the Chinese context, the expressions of *resisting* and *criticizing* are always expressed euphemistically, making them hard to distinguish. The researchers likely needed to be involved in the context to understand them. It seemed that they reflected more on the rejection of participants, confusion, and uncertainty about the TRA reform.

**FIGURE 1:**
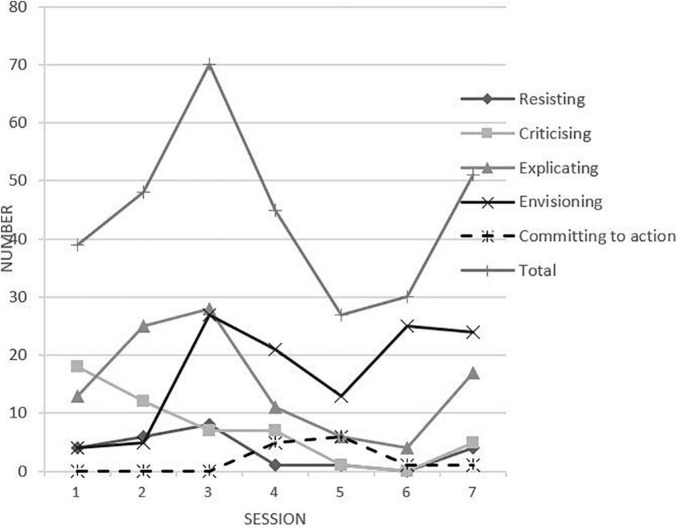
The transformative agency in every session.

As Figure 1 shows, in the earlier two sessions, the expressions of transformative agency focused more on *criticizing* and *explicating*. The *criticizing* gradually declined since Session 1, but *explicating*, *resisting*, and *envisioning* gradually increased. The high point was in Session 3, a turning point from *explicating* to *envisioning*. Before Session 3, the expressions of *explicating* were more frequent than *envisioning*, while *envisioning* was more frequent afterward. At the same time, the frequency of *resisting* began to decline. The transformative agency was weak in the middle and later sessions, reading a low point at Session 5 because the teachers in this session spent more time demonstrating a micro-class example instead of discussing it. It seemed there was a developmental movement from *resisting* and *criticizing* toward textitenvisioning.

### The Expression of Transformative Agency in an Activity System

The discussions among the teachers and researchers in the Change Laboratory focused on reform based on making the TRA more efficient. According to activity theory (Engeström, 2015), this reform, as a human activity, has six components. The *subject* is the teachers whose perspective is adopted. Teachers use *tools*, such as school resources and self-study guides, to achieve the *object* of improving the efficiency of the reform activity. The changes in TRA are planned and implemented by a *community* of teachers within a school. In the Change Laboratory, this community was expanded to include experienced teachers invited from other schools and pedagogical experts from universities. The *rules* under which reform is enacted include formal and informal conventions, guidelines, contracts, laws, and other societal norms about education. The rules about TRA are now required under the New Curriculum Reform. The division of labor refers to how the teachers share the responsibilities of enacting these reforms.

How often was transformative agency expressed about these six elements of the activity system? The expressions of transformative agency were most common concerning tools: the number was 152 out of 332. The expressions related to other key elements of the activity system were made with the following frequency, in decreasing order: community (61), object (53), subject (40), rules (20), and division of labor (6).

**FIGURE 2:**
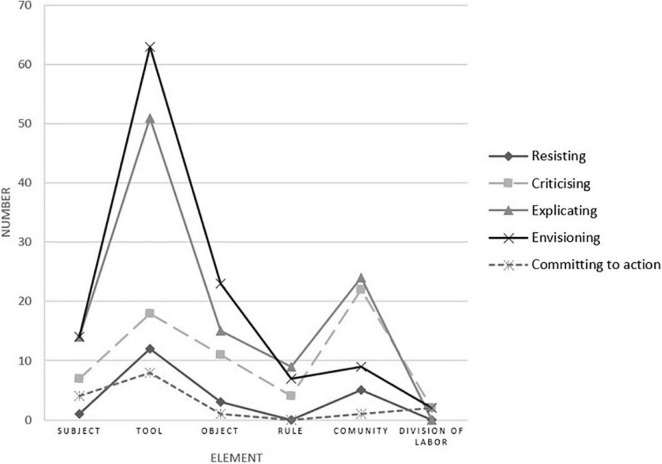
The expression of transformative agency in the activity system.

This information was also examined in terms of how often each of the six types of transformative agency (Haapasaari et al., 2016) was expressed about the six elements of the activity system. As shown in Figure 2, *envisioning* and *explicating* were much more frequently directed at the tools than at other elements of the activity. The limited expressions of *criticizing* were mainly about the community, which was relatively hard to change. Although participants had some complaints and criticisms about the community, they were still hoping to get help from their colleagues, school leaders, and teachers from other schools to reform the current TRA to be more efficient.

### The Uneven Contribution of Participants

The transformative agency differed across the teacher participants. Based on the analysis of its expressions, the participants could be divided into three groups according to the number of speaking turns, namely, (1) Highly active participants, marked with * in Table 4. Of these, Lee was the most active. She contributed to the largest number of change initiatives. (2) Moderately active participants were Ting, Chen, and Lin. (3) Low active participants were Xin, Fu, Huan, Ming, Lin, and Yi. Table 4 shows that there is a big gap in the number of various expressions among the different types of teachers. Some teacher participants had many experiences and suggestions to share, but after one or two sessions others preferred to be silent and listen to others.

**TABLE 4:** The number of transformative agency expressions for different participants.

Teacher	Resisting *f(%)*	Criticizing *f(%)*	Explicating *f(%)*	Envisioning *f(%)*	Committing to action *f(%)*	Total *f(%)*
Lee*	6 (27.27)	2 (4.08)	33 (32.04)	71 (56.80)	6 (54.55)	118 (38.06)
Hui*	2 (9.09)	14 (28.57)	30 (29.13)	14 (11.20)	4 (36.36)	64 (20.65)
Ting	3 (13.64)	6 (12.24)	5 (4.85)	14 (11.20)	0	28 (9.03)
Chen	3 (13.64)	6 (12.24)	5 (4.85)	13 (10.40)	0	27 (8.71)
Lin	4 (18.18)	9 (18.37)	3 (2.91)	2 (1.60)	1 (9.09)	19 (6.13)
Xin	1 (4.55)	1 (2.04)	8 (7.77)	3 (2.40)	0 (0)	13 (4.19)
Fu	2 (9.09)	1 (2.04)	9 (8.74)	0	0	12 (3.87)
Huan	0 (0)	4 (8.16)	4 (3.88)	3 (2.40)	0	11 (3.55)
Ming	0	1 (2.04)	3 (2.91)	5 (4.00)	0 (0.00)	9 (2.90)
Yi	1 (4.55)	5 (10.20)	3 (2.91)	0	0	9 (2.90)

*Means that the teacher was a highly active participant.

We found that the teacher participants with richer experiences were much more likely to share their opinions. The most typical representative was Lee, the English teacher. She had 6 years of teaching experience. The type she expressed was mainly *envisioning*, a type that was expressed with much less frequency by the low active participants.

## Discussion

### The Expression of Transformative Agency in Chinese Culture

In our Change Laboratory, the expression of the transformative agency was quite different from previous studies. In previous research on transformative agency, *criticizing* was the most frequently expressed type (Haapasaari et al., 2016), and *resisting* the management or the interventionist is also common in the discourse of teachers (Sannino, 2010). By contrast, in our study, *criticizing* and *resisting* were the least common types. *Explicating* was also of relatively low frequency and *envisioning* was of relatively high frequency.

Several cultural factors might explain these results. In the Chinese collectivist culture, people are not likely to express much criticism and resistance, which may offend the interests of others and negatively affect interpersonal relationships. Chinese culture is relation-based, and in most cases, it is difficult to “only attack the problem, but not the person.” People tend to see the topic of communication as intrinsic to the person, if the issue is attacked, so is the person. To avoid interpersonal conflicts, people often choose to be silent during a conversation. Communication in Chinese culture is “listening-centered”; that is, listening is more important than speaking. Mostly, silence is a sign of listening and thinking carefully.

The theory of high- and low-context communication proposed by Hall (1976) highlights this phenomenon by pointing to Chinese culture as a typical high-context communication culture. In such a communication culture, the atmosphere and context are very important when people communicate, and they often also need to pay attention to the meaning expressed by non-verbal cues. Once people are understood, they can relate to others and feel they are in the same community. That is also what we found in our study. When the education experts were explaining the micro-class example, the teachers listened carefully, nodded, and took notes carefully. Then some teachers explained the opinion of an expert in their own words, asking the opinion of an expert to make sure that they really understood it. Whether it is an education expert or a teacher, when explaining, he or she will look at the eyes of other people and other information to see if they understood and then decide if they need further explanation. The result suggests that compared with *criticizing* and *resisting*, *explicating* is more important for *envisioning* the transformative process in Chinese culture.

### The Expressions of Transformative Agency in the Activity System

The expressions of transformative agency of teachers focused mainly on tools, accounting for 49.4% of the total expressions. The ratio was significantly higher than other elements of the system in the Laboratory. This is similar to the results proposed by Haapasaari et al. (2016) in which the most discussed topics related to the transformative agency were the subject (26%) and tools (22%) of the activity system. In our study, all expression types of the transformative agency had a focus on the tools, especially in the cases of *explicating* and *envisioning.* The result of our study is consistent with that of Vänninen et al. (2015).

In the Change Laboratory, the teachers started systematically communicating about TRA issues and tried exploring the ways to solve these problems. The transformative agency of participants focused more on the *micro-class example* tool, a way of showing teaching, which was first proposed by the researchers in Session 3. The *micro-class example* helps teachers to capture the problem with a simple idea that can be transformed and expanded into new forms of practice, overcoming the gap between theoretical conception and practice. Participants discussed the application difficulties of the tool. In the beginning, teachers did not know much about it, and there was some criticism and resistance. However, after the attempts in Session 4 and Session 5, teachers had some new ideas of their own about the *micro-class example*. Of course, the final practical application of the *micro-class example* is further modified by teachers, which is not the same as what the researchers originally proposed.

Tools help produce qualitative transformations both in individuals and in their environments (Jonassen and Rohrer-Murphy, 1999; Engeström and Sannino, 2010; Engeström, 2015). For the transformative agency to emerge in an intervention, the subjects must invest in agentive initiatives and volitional actions to transform their activities (Vänninen et al., 2015). In our research, under the influence of culture, people tried to avoid interpersonal conflict in the discussion. The emergence of new tools caused the participants to weigh their desire to change their intentions against their fear of encountering conflicts with others. Discussing tools has much less risk. For both teachers and school leaders, changing tools may bring desirable outcomes for the TRA.

### Individual Differences in Transformative Agency

The development of agency is actively taken up by each individual (Stetsenko, 2019). In the Change Laboratory, although the researchers would encourage everyone to actively participate in the discussion, there were still differences in individual participation. Among the highly active participants, Lee had more teaching experience and insights to share since she has been a teacher for many years, creating a power differential between her and other teacher participants. Hui was the second most active participant (see Table 4). Before entering this school, she worked in another school for 2 years. During the discussion, she shared a lot of previous work experience. Unlike the activity of Lee, her most important expression of change was “interpretation,” which compared past positive experiences with current activities.

Among the low-active participants, Ming was a fifth-grade English teacher. Her biggest concern was about the community of the TRA. She also referred to the “apprenticeship system” and “shadow learning” as needing help from the community to transform TRA. These points were related to the identity of Ming. She was a new teacher who had just been teaching for 1 year, often encountered new situations that were difficult to cope with, and hoped to get targeted guidance. Fu was very motivated to participate in the early stage, but since he was the only physical education teacher in the whole event, there was no discussion between the teachers with the same subject and his feedback did not resonate with the other discussants. He was even absent from some of the later meetings and felt irrelevant to the change activities.

The concept of legitimate peripheral participation provides a way to speak about crucial relations between newcomers and old-timers, and about their activities, identities, artifacts, knowledge, and practice (Lave, 1991). In group activities, there are bound to be individuals with different levels of activity, but their influence and interaction with each other is the way to establish contact with each other (Ryan and Deci, 2000; Villegas-Reimers, 2003; Thurlings and den Brok, 2017). During the discussion, experienced participants often have more active voices and are more likely to act as problem-solvers. They are proponents and listeners to others who talk about the problem. The activity will be less effective if these experienced teachers do not remain fully engaged in all sessions.

Disparities in the level of participation need particular attention. On the one hand, we should value the participants who have certain experience and are willing to actively try, and encourage them to drive everyone to participate in the change. On the other hand, we should find ways to encourage those who prefer silence to speak up. If the change activity can well meet the needs of individuals and give timely feedback, it will improve the transformative agency of participants. By creating an open and free team atmosphere in the Change Laboratory, participating teachers can freely pursue issues most relevant to them, understand individual needs and expectations of each other, and then try their best to meet their personal development needs in the activities, thus increasing their transformative agency.

## Data Availability Statement

The original contributions presented in the study are included in the article/supplementary material, further inquiries can be directed to the corresponding authors.

## Author Contributions

CD contributed to the project administration, writing the original draft, methodology, and data curation. XZ helped in writing the original draft, investigation, and data curation. QM contributed to the conceptualization, methodology, validation, and funding acquisition. JH contributed to the supervision, writing, reviewing, and editing. All authors contributed to the article and approved the submitted version.

## Conflict of Interest

The authors declare that the research was conducted in the absence of any commercial or financial relationships that could be construed as a potential conflict of interest.

## Publisher’s Note

All claims expressed in this article are solely those of the authors and do not necessarily represent those of their affiliated organizations, or those of the publisher, the editors and the reviewers. Any product that may be evaluated in this article, or claim that may be made by its manufacturer, is not guaranteed or endorsed by the publisher.
